# Neighborhood- and Patient-Level Socioecological Determinants of Health Assessed Before Major Surgery

**DOI:** 10.1001/jamanetworkopen.2025.32854

**Published:** 2025-09-19

**Authors:** Kurt S. Schultz, Samantha M. Linhares, Elizabeth L. Godfrey, Uday Dhanda, Zane J. Hellmann, Hannah W. Van Dusen, Daniel I. Chu, Ira L. Leeds

**Affiliations:** 1 Division of Colon & Rectal Surgery, Department of Surgery, Yale School of Medicine, New Haven, Connecticut; 2 Division of GI Surgery, Department of Surgery, University of Alabama at Birmingham, Birmingham

## Abstract

This cross-sectional study of US adults undergoing elective major surgery examines the correlation of neighborhood-level and patient-level socioecological determinants of health for projecting health outcomes.

## Introduction

Socioecological determinants of health (SEDOH) influence surgical outcomes.^1^ Due to their accessibility, neighborhood-level indices are utilized as proxies for patient-level SEDOH status. However, neighborhood-level indices are prone to ecological fallacy.^2^ This study evaluated the correlation between neighborhood- and patient-level SEDOH domains before major surgery.

## Methods

This study was a cross-sectional analysis of a prospectively maintained SEDOH assessment involving adults undergoing elective major surgery across 2 hospitals and 3 surgical services from July 2023 to January 2025. Patients’ residential addresses were geocoded to the US census tract level and block group for the Social Vulnerability Index (SVI) and the Area Deprivation Index (ADI), respectively. Patients with missing or nongeocodable addresses were excluded.

The SEDOH-88 survey served as a patient-level measure (with a higher score indicating higher risk).^3^ This survey was administered to patients within 2 weeks before their surgery through structured, researcher-led interviews. Missing responses were treated as nonendorsement of the specific SEDOH item (eMethods in Supplement 1; eTable in Supplement 2).

Sex, gender, race, and ethnicity were self-identified by patients and assessed due to suspected SEDOH disparities. Data were analyzed using receiver operating characteristic curves (ROC), multivariable linear regression, and the Spearman rank correlation coefficient. The SEDOH-88 score was treated as a binary variable for the ROC curves (with high defined as above the 75th percentile) and as a continuous outcome for the regression and correlation analyses. Age, comorbidities, and the hospital site were fixed effect covariates in the regression.

Categorical variables were reported as percentages and compared using χ^2^ and Fisher exact tests. Continuous variables were reported as medians and interquartile ranges (IQR) and compared using the Kruskal-Wallis test. Two-tailed CIs were set at 95% and significance levels were set at *P* < .05 (Bonferroni correction for multiple comparisons). Statistical analysis was performed in R version 4.3.1 (R Project for Statistical Computing). The Yale University institutional review board approved the study, and informed verbal consent was obtained from participants. This study followed the Strengthening the Reporting of Observational Studies in Epidemiology (STROBE) reporting guideline.

## Results

Of 345 patients contacted, 234 (67.8%) agreed to participate, 130 (55.6%) self-identified as women; 8 participants (3.4%) identified as African American or Black, 2 (0.9%) American Indian or Alaskan Native, 4 (1.7%) Asian, 216 (92.3%) White, and 4 (1.7%) with unspecified race; 118 participants (7.7%) identified as Hispanic or Latine (Table). There were no missing geographic identifiers.

**Table.** Demographics, Socioecological Determinants of Health, Clinical Characteristics, and Outcomes of Patients Undergoing Elective Major Surgery Within a Statewide Health System

Characteristics	No. (%)				*P* value
	Overall (n = 234)	Thoracic (n = 60)	Abdominal Oncology (n = 45)^a^	Colorectal (n = 129)	
**Neighborhood-level indices^b^**					
ADI, median (IQR), percentile	31.5 (20.3-45.0)	24.0 (18.0-41.0)	36.0 (24.0-50.0)	33.0 (22.0-46.0)	.09
SVI, median (IQR), percentile					
Overall	0.29 (0.12-0.58)	0.23 (0.08-0.63)	0.31 (0.20-0.48)	0.28 (0.12-0.59)	.73
SVI-1 (socioeconomic)	0.27 (0.13-0.54)	0.24 (0.12-0.57)	0.28 (0.17-0.46)	0.28 (0.13-0.56)	.93
SVI-2 (household)	0.37 (0.20-0.60)	0.34 (0.26-0.56)	0.52 (0.31-0.73)	0.35 (0.17-0.58)	.09
SVI-3 (race and ethnicity)	0.33 (0.19-0.53)	0.33 (0.21-0.53)	0.27 (0.15-0.47)	0.37 (0.22-0.53)	.24
SVI-4 (housing and transport)	0.35 (0.19-0.56)	0.36 (0.11-0.70)	0.34 (0.19-0.54)	0.35 (0.20-0.55)	.97
**Patient-level measure^b^**					
SEDOH-88, median (IQR)	5 (3-8)	5 (4-8)	5 (3-7)	5 (3-7)	.48
Age, median (IQR), y	64.0 (51.0-71.0)	68.5 (58.8-73.3)	58.0 (50.0-66.0)	63.0 (50.0-71.0)	.005
Sex assigned at birth					
Female	131 (56.0)	28 (46.7)	26 (57.8)	77 (59.7)	.24
Male	103 (44.0)	32 (53.3)	19 (42.2)	52 (40.3)	
Self-identified gender					
Woman	130 (55.6)	28 (46.7)	26 (57.8)	76 (58.9)	.47
Man	103 (44.0)	32 (53.3)	19 (42.2)	52 (40.3)	
Nonbinary	1 (0.4)	0	0	1 (0.8)	
Self-identified race					
African American or Black	8 (3.4)	1 (1.7)	1 (2.2)	6 (4.7)	.21
American Indian or Alaskan Native	2 (0.9)	1 (1.7)	0	1 (0.8)	
Asian	4 (1.7)	1 (1.7)	2 (4.4)	1 (0.8)	
White	216 (92.3)	54 (90.0)	41 (91.1)	121 (93.8)	
Not specified^c^	4 (1.7)	3 (5.0)	1 (2.2)	0	
Self-identified ethnicity					
Hispanic or Latine	18 (7.7)	4 (6.7)	3 (6.7)	11 (8.5)	.87
Non-Hispanic or Latine	216 (92.3)	56 (93.3)	42 (93.3)	118 (91.5)	
Primary insurance coverage					
Government	133 (56.8)	40 (66.7)	19 (42.2)	74 (57.4)	.12
Private	99 (42.3)	20 (33.3)	25 (55.6)	54 (41.9)	
Uninsured	2 (0.9)	0	1 (2.2)	1 (0.8)	
High-risk medical comorbidities^d^					
BMI ≥35	35 (15.0)	7 (11.7)	7 (15.6)	21 (16.3)	.70
Diabetes	41 (17.5)	14 (23.3)	8 (17.8)	19 (14.7)	.35
Functional status	3 (1.3)	0	0	3 (2.3)	.29
COPD	29 (12.4)	16 (26.7)	1 (2.2)	12 (9.3)	<.001
Ascites	3 (1.3)	0	3 (6.7)	0	.002
Heart failure	1 (0.4)	0	0	1 (0.8)	.66
Disseminated cancer	49 (20.9)	10 (16.7)	27 (60.0)	12 (9.3)	<.001
Immunotherapy	25 (10.7)	2 (3.3)	5 (11.1)	18 (14.0)	.09
Bleeding disorders	32 (13.7)	12 (20.0)	7 (15.6)	13 (10.1)	.17
Oxygen support	3 (1.3)	1 (1.7)	0	2 (1.6)	.70
Fall in the last 6 mo	10 (4.3)	3 (5.0)	2 (4.4)	5 (3.9)	.94
Dementia	4 (1.7)	2 (3.3)	1 (2.2)	1 (0.8)	.43
High-risk medical comorbidity^e^					
≥1 comorbidity	148 (63.2)	40 (66.7)	39 (86.7)	69 (53.5)	<.001
No. of high-risk medical comorbidities					
0	86 (36.8)	20 (33.3)	6 (13.3)	60 (46.5)	.005
1	91 (38.9)	21 (35.0)	25 (55.6)	45 (34.9)	
2	33 (14.1)	11 (18.3)	7 (15.6)	15 (11.6)	
≥3	24 (10.3)	8 (13.3)	7 (15.6)	9 (7.0)	
Surgical indication					
Benign disease	78 (33.3)	6 (10.0)	5 (11.1)	67 (51.9)	<.001
Cancer (or suspected)	156 (66.7)	54 (90.0)	40 (88.9)	62 (48.1)	
Surgical approach					
Open	48 (20.5)	12 (20.0)	19 (42.2)	17 (13.2)	<.001
Laparoscopic and thoracoscopic	60 (25.6)	10 (16.7)	13 (28.9)	37 (28.7)	
Robotic	120 (51.3)	38 (63.3)	12 (26.7)	70 (54.3)	
Converted to open	6 (2.6)	0	1 (2.2)	5 (3.9)	
30-d readmission	23 (9.8)	13 (21.7)	7 (1.6)	3 (2.3)	.20
90-d mortality	6 (2.6)	2 (3.3)	2 (4.4)	2 (1.6)	.52

Abbreviations: ADI, Area Deprivation Index; BMI, body mass index (calculated as weight in kilograms divided by height in meters squared); COPD, chronic obstructive pulmonary disease; SEDOH-88, Socioecological Determinants of Health-88; SVI, Social Vulnerability Index.

^a^ Indications for abdominal oncology procedures were hepatopancreatobiliary cancers, peritoneal surface malignant neoplasms, and sarcomas.

^b^ SVI has 4 individual themes: socioeconomic status, household composition and disability, minority status and language, and housing type and transportation. SEDOH-88 is a composite questionnaire of validated survey instruments administered to patients preoperatively.

^c^ The race category of “not specified” comprised patients who self-identified as Hispanic or Latine for ethnicity but did not report a race.

^d^ No patients had the following comorbidities: ventilator dependence, dialysis, blood transfusion, and sepsis.

^e^ High-risk medical comorbidity was a binary variable. Patients with at least 1 of the individual comorbidities listed in this table were classified as having a high-risk medical comorbidity.

SVI and ADI showed poor discrimination with the SEDOH-88 score, with an area under the curve (AUC) of 0.667 (95% CI, 0.588-0.746; *P* < .001) and 0.626 (95% CI, 0.539-0.713; *P* = .005), respectively. The 4 SVI themes also discriminated poorly with the SEDOH-88 score: AUC was 0.651 (95% CI, 0.570-0.733) for theme 1, 0.584 (95% CI, 0.495-0.674) for theme 2, 0.651 (95% CI, 0.564-0.737) for theme 3, and 0.668 (95% CI, 0.587-0.749) for theme 4. In multivariable linear regression, SEDOH-88 was associated with SVI (β = 3.80; 95% CI, 1.51-6.08; *P* = .001) but not with ADI (β = 0.03; 95% CI, −0.002 to 0.07; *P* = .07). Neither SVI (ρ = 0.195; *P* = .003) nor ADI (ρ = 0.139; *P* = .03) correlated well with SEDOH-88 (Figure).

**Figure.**
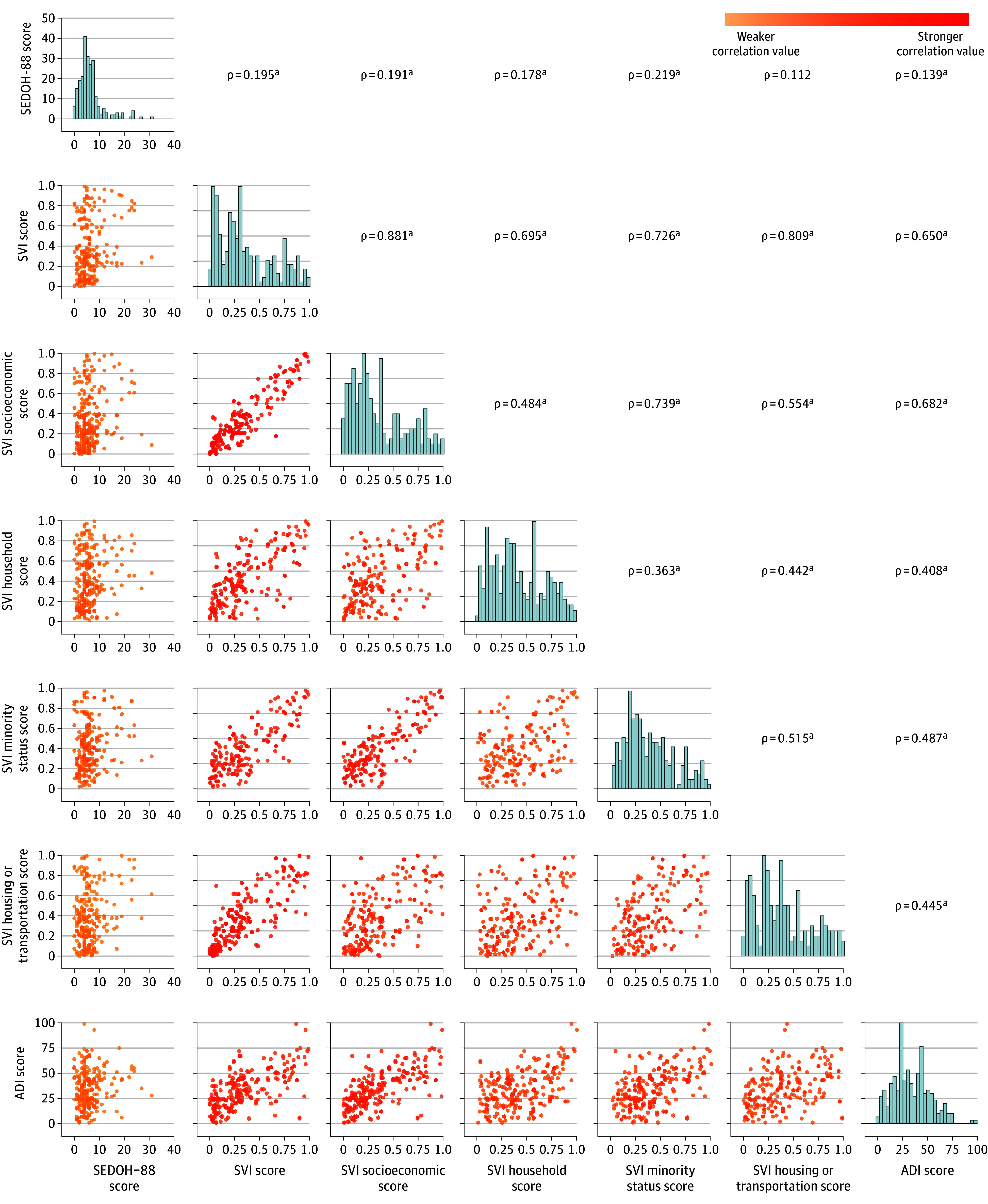
Scatterplot Matrix of Neighborhood- and Patient-Level Socioecological Scores ADI indicates Area Deprivation Index; SEDOH-88, Socioecological Determinants of Health-88; SVI, Social Vulnerability Index. The color scheme of the lower triangle represents the gradation of correlation between the scores, corresponding to the pairwise relationships derived from the Spearman rank correlation coefficient. Darker colors indicate stronger correlation, while lighter colors indicate weaker correlation. The upper triangle displays the Spearman rank correlation coefficient (ρ). Diagonal panels display univariate histograms of each variable. SEDOH-88 is a composite questionnaire of validated survey instruments administered to patients preoperatively. SVI has 4 individual themes: socioeconomic status, household composition and disability, minority status and language, and housing type and transportation. ^a^Denotes a significant result (*P* < .05).

## Discussion

This study revealed a limited correlation between neighborhood-level indices and a patient-level SEDOH assessment conducted before major surgery. These results are consistent with nonsurgical studies that describe the limited capability of population-level data to predict patient-level SEDOH domains.^4,5^

In March 2025, the Centers for Medicare & Medicaid Services rescinded their endorsement of assessing patient-level SEDOH status.^6^ However, our study cautions against relying solely on neighborhood-level characteristics and proposes a model that integrates psychosocial risk assessment into preoperative care. Our findings suggest that policy should include patient-level assessments to target vulnerabilities more accurately.

A limitation of this study is the potential for residual confounding, as the number of covariates included in our regression model was restricted to avoid loss of precision and minimize sparse-data bias. Future multi-institutional studies should evaluate the relative and combined predictive value of patient- and neighborhood-level factors on surgical outcomes.

## Notes

Supplement 1.
eMethods.

Supplement 2. **eTable.** Adapted SEDOH-88 Survey

Supplement 3.
Data Sharing Statement
