# Supplementary material for: Neighborhood- and Patient-Level Socioecological Determinants of Health Assessed Before Major Surgery
Source: JAMA Netw Open. 2025 Sep 19;8(9):e2532854. doi: 10.1001/jamanetworkopen.2025.32854 (PMC12449718; doi:10.1001/jamanetworkopen.2025.32854)
Supplement: Supplement 1. — eMethods. [file jamanetwopen-e2532854-s001.pdf]

Supplemental Online Content

Schultz KS, Linhares SM, Godfrey EL, et al. Neighborhood- and patient-level socioecological determinants of health assessed before major surgery. *JAMA Netw Open.* 2025;8(9):e2532854. doi:10.1001/jamanetworkopen.2025.32854

eMethods.

This supplemental material has been provided by the authors to give readers additional information about their work.

© 2025 Schultz KS et al. *JAMA Network Open*.

eMethods. Patient-level socioecological risk measure.

The SEDOH-88 survey is a comprehensive, patient-reported socioecological assessment tool developed by The Chu Lab at the University of Alabama at Birmingham.[1] The tool has 88 questions across 31 socioecological determinants of health domains. The patient-level socioecological risk measure used in this study was adapted from the SEDOH-88 survey. eTable 1 outlines the domains, questions, and scoring system for this study's patient-level measure. The survey's minimum and maximum scores are 0 and 62 points, respectively. Missing responses were treated as non-endorsement of the specific SEDOH item for binary and checklist items (i.e., no points added to the overall SEDOH score). For domains assessed using multi-item scales (e.g., social support), missing items were handled using pairwise deletion, also known as available-case analysis, which resulted in partial domain scores based on the available data. This approach assumes that missing responses indicate no vulnerability, reflecting a conservative imputation strategy that minimizes false positives. These methods for handling missingness were applied consistently across all SEDOH domains.

References

1. Smith B, Smith BP, Hollis RH, et al. Development of a comprehensive survey to assess key socioecological determinants of health. *Surgery*. Apr 2024;175(4):991-999. doi:10.1016/j.surg.2023.11.011

© 2025 Schultz KS et al. *JAMA Network Open*.
