# Supplementary material for: Neighborhood- and Patient-Level Socioecological Determinants of Health Assessed Before Major Surgery
Source: JAMA Netw Open. 2025 Sep 19;8(9):e2532854. doi: 10.1001/jamanetworkopen.2025.32854 (PMC12449718; doi:10.1001/jamanetworkopen.2025.32854)
Supplement: Supplement 3. — Data Sharing Statement [file jamanetwopen-e2532854-s003.pdf]

Data Sharing Statement

Data
Data available: No

Additional Information
Explanation for why data not available: Neighborhood level indices are publicly available already. SEDOH-88 self reported questionnaires data are sensitive topics for many patients and the IRB consent to participate did not involve the potential for their data to be shared without aggregation first.
